# Altered cognition in patients following pulmonary embolism

**DOI:** 10.3389/fmed.2025.1746780

**Published:** 2026-01-08

**Authors:** Shuying Jia, Chunyan Sun, Xingquan Xiong, Xin Liu, Fang Wang, Yuyan Liu, Jinling Li, Lei Zhao

**Affiliations:** 1 School of Gongli Hospital Medical Technology, University of Shanghai for Science and Technology, Shanghai, China; 2 Shanghai Pulmonary Hospital, School of Medicine, Tongji University, Shanghai, China; 3 Department of Pulmonary and Critical Care Medicine, Shanghai Pudong New Area Gongli Hospital, Shanghai, China

**Keywords:** cognitive dysfunction, Mini-Mental State Examination, Montreal Cognitive Assessment, pulmonary embolism (PE), risk factors

## Abstract

**Background:**

Acute pulmonary embolism (PE) is a critical disease and often leads to high mortality and morbidity. Growing studies have identified the diagnosis and treatment of PE, but the cognitive situation of PE patients remains unclear. This study investigates cognitive the status in patients with PE and proposes potential lung-brain interaction mechanisms and clinical implications.

**Methods:**

We enrolled 400 adult patients aged > 18 years, diagnosed with acute PE and 91 matched healthy controls at Shanghai Pulmonary Hospital between May 2018 and December 2024. Objective cognitive status was assessed using the Chinese versions of the Mini-Mental State Examination (MMSE) and the Montreal Cognitive Assessment (MoCA), and total scores and subscores for each cognitive domain were calculated for each patient. We summarized the baseline characteristics, relevant laboratory tests, recurrence, and risk stratification features of the PE patients, compared the total and sub-item scores of the MMSE and MoCA, performed univariate and multivariate logistic regression analyses, and employed a Cox proportional hazards model to analyze prognostic factors in the PE cohort.

**Results:**

Pulmonary embolism patients had significantly lower total MMSE and MoCA scores than healthy controls, with declines across sub-items of orientation, Attention/Calculation (A&C), Visuospatial transformation (V-S total), and Delayed recall, indicating widespread cognitive impairment; patients with high risk and recurrence had even lower MMSE and MoCA scores (*p* < 0.05). Kaplan-Meier (KM) analysis demonstrated that patients with lower scores had higher recurrence rates and higher PE risk stratification. The ROC curve indicates that combining MMSE < 27.5 and MoCA < 25.5 can significantly improve the predictive efficacy for the occurrence of PE.

**Conclusion:**

A significant decrease in the MMSE and MoCA cognitive function assessment scales is associated with a higher occurrence of PE and worse disease prognosis. Therefore, it is necessary to conduct cognitive function screening for patients with pulmonary embolism and to intervene as early as possible. Moreover, this finding further suggests that patients with PE constitute a potentially important group for cognitive rehabilitation, providing a valuable reference for the screening of populations in need of neurorehabilitation.

## 1 Introduction

Acute pulmonary embolism (PE), caused by endogenous or exogenous thrombosis in the pulmonary arterial trunk or its branches, is a cardiovascular emergency (1–3). PE is the third most common acute cardiovascular syndrome globally, following myocardial infarction and stroke. The incidence of PE worldwide is approximately 39–115 cases per 100,000 the population per year, with around 1 million deaths attributed to PE annually. In China, the incidence of PE is on the rise, and the in-hospital mortality rate for hospitalized PE patients is 3.9% (4). Both globally and in China, PE exhibits epidemiological characteristics of high incidence and high mortality. Examination methods include computerized tomographic pulmonary angiography, planar or single-photon emission computed tomography (SPECT).

Acute PE is associated with a certain rate of recurrence, followed by prolonged anticoagulant therapy. The common clinical manifestations includes dyspnea, chest pain, cough or syncope (5, 6). In addition to the aforementioned symptoms, a case report described a 64-years-old female patient who was ultimately diagnosed with acute PE and Patent Foramen Ovale (PFO), presenting with persecutory delusions and abnormal behavior as initial manifestations. Her MMSE score was only 22/30 (7). Moreover, studies have indicated that the risk of neurological complications in patients with PE is 3.5 times higher than that in patients without PE (8). Therefore, whether the occurrence of this disease is associated with cognitive impairment requires further comprehensive exploration.

Some evidence indicates that chronic cardiovascular and pulmonary diseases (such as hypertension, atherosclerosis, diabetes, chronic obstructive pulmonary disease, acute respiratory distress syndrome) are associated with cognitive impairment due to inflammation, oxidative stress, and mitochondrial dysfunction q (9–11). In patients with COPD, elevated levels of inflammatory markers such as serum amyloid A (SAA) are significantly associated with cognitive impairment, suggesting that the inflammatory pathway plays an important role in the lung-brain axis interaction (12). Studies have revealed that the incidence of cognitive impairment is relatively high among patients with heart failure, and the severity of cognitive impairment tends to increase with the worsening of heart failure (13). For instance, patients with higher New York Heart Association (NYHA) functional classifications exhibit more pronounced cognitive impairments. Moreover, cerebral hypoperfusion caused by heart failure is one of the primary mechanisms underlying cognitive impairment. In heart failure with reduced ejection fraction (HFrEF), cerebral blood flow can be reduced by approximately 14%–30% (14). Although some studies have found a close relationship between cardiopulmonary diseases such as COPD and heart failure and cognitive impairment, to date, there has been no systematic and detailed report revealing the specific association between pulmonary embolism and cognitive impairment.

In patients with PE, dyspnea or loss of consciousness can lead to hemodynamic changes, which are associated with cognitive impairment related to inflammation, oxidative stress, and mitochondrial dysfunction (15–17). Unlike the long-term chronic effects associated with COPD and chronic heart failure, pulmonary artery obstruction leads to a sharp decline in blood oxygenation, causing systemic hypoxemia and insufficient oxygen supply to brain tissue. Severe hypoxia can induce cerebral edema and disruption of the blood–brain barrier, resulting in persistent neurological deficits. Decreased pulmonary function reduces cerebral oxygen supply, impairs vascular endothelial function, and lowers cerebral blood flow, ultimately exacerbating cerebral ischemia and promoting white matter lesions and lacunar infarcts. Moreover, PE activates a systemic inflammatory cascade, with inflammatory cytokines (such as IL-6 and TNF-α) able to cross the blood–brain barrier, inducing neuroinflammation and accelerating neuronal damage (18). It has been reported that approximately 45% of patients with PE have elevated levels of brain natriuretic peptide (BNP), which further suggests that brain injury may occur in PE patients, leading to cognitive impairment in certain brain regions (19).

Timely identification of cognitive dysfunction and clarification of its potential influencing factors, particularly those related to pulmonary embolism, are of utmost importance. While the clinical community is increasingly recognizing the cognitive status of PE patients, there is still a lack of large-scale, detailed, and systematic cognitive assessments to elucidate cognitive function and its correlates in these patients. Therefore, this study aims to explore the correlation between pulmonary embolism and cognitive impairment, providing reference value for cognitive function screening and early intervention in clinical PE patients. It also provides further clinical evidence for studying the relationship between the lungs and the brain.

## 2 Materials and methods

A retrospective cohort study data were derived from May 2018 to December 2024 by pulmonary department center which focused on pulmonary circulation diseases in Shanghai Pulmonary Hospital. In this study, the diagnosis of pulmonary embolism (PE) was based on the 2019 European Society of Cardiology (ESC) guidelines. These guidelines recommend the use of clinical or preoperative probability assessment, D-dimer testing, computed tomography pulmonary angiography (CTPA), ventilation/perfusion scanning, or lower limb ultrasound for diagnosis (20–23).

Patients eligible for inclusion in this study must meet at least one of the following criteria: a positive Wells score, a revised Geneva score (RGS), or a simplified RGS. The inclusion criteria also include the presence of symptoms such as dyspnea, pleuritic chest pain, syncope, or new-onset fatigue. Additionally, patients with a heart rate exceeding 100 beats per minute or an abnormal oxygen saturation level below 95% at sea level that cannot be explained by other causes were included. The information collected includes clinical variables, educational level, biochemical markers, comorbidities, clinical signs and symptoms, risk stratification, and recurrence status. Risk stratification is assessed using the Simplified Pulmonary Embolism Severity Index (sPESI), where a score ≥ 1 indicates moderate to high risk and <1 indicates low risk. All patients underwent baseline assessments, including clinical evaluations and laboratory tests. Recent surgery is defined as having undergone a surgical procedure within 2 months before the diagnosis of pulmonary embolism.

The patients with insufficient data, APE of non-thrombus origin (fat or tumor emboli), severe mental illness, alcoholism, and those using psychotropic medications and patients with active cancer (newly diagnosed cancer or metastatic cancer) or thrombophilia were excluded. We confirmed the recurrence of PE in patients through imaging modalities such as computed tomography pulmonary angiography (CTPA), pulmonary angiography, and ventilation/perfusion lung scans (V/Q scans), as well as relevant biochemical markers (such as D-dimer and CK-MB) and clinical symptoms, excluding suspected events without imaging confirmation.

All patients were followed up at 1 month, 3 months, 6 months, the first year, second year, and fifth year after discharge, either through outpatient visits or telephone follow-ups. The follow-up focused on assessing the relief of symptoms, as well as the adherence to and tolerability of anticoagulation therapy. The median follow-up duration was 35 months. Additionally, during the study period, some patients were not newly diagnosed with PE. Instead, they underwent cognitive function scale assessments after admission. Therefore, there is a temporal discrepancy between the time of cognitive evaluation and the time of diagnosis.

Control group subjects are age- and gender-matched, subjects without PE, chronic lung disease, and neurological disorders were included in the control group. This study was approved and supervised by the ethical committee of the Shanghai Pulmonary Hospital (approval number: K21-316). Written informed consent was obtained from all subjects in this study. A total of 400 patients diagnosed with PE were included in this study.

The cognitive function of the participants was assessed using the Mini-Mental State Examination (MMSE) and the Montreal Cognitive Assessment (MoCA) scales. The MMSE is a widely used global cognitive screening tool that covers five domains: orientation, memory, language, recall, and attention and calculation. The Montreal Cognitive Assessment (MoCA), developed by the Nasreddine team in 2005, is a rapid cognitive screening tool that includes eight cognitive domains: attention and concentration, executive function, memory, language, visuospatial skills, abstract thinking, calculation, and orientation. Since the MoCA scale is closely related to the educational level of the subjects, when using the MoCA scale, patients with PE who have an educational duration of less than 12 years should be awarded an additional point in the total score to correct the bias caused by differences in educational level. Both scales use a 30-point scoring system, with higher scores indicating better cognitive function.

The MMSE and MoCA scales are widely used cognitive function screening tools in clinical practice. Although the MMSE is extensively applied, it has certain limitations in screening for mild cognitive impairment. It primarily focuses on basic cognitive functions and has relatively low sensitivity for early cognitive dysfunction. However, it demonstrates high sensitivity for moderate to severe cognitive impairments. Therefore, the combined use of these two scales for assessment can complement each other’s scope of involvement.

Mini-Mental State Examination and MoCA scores were compared by using *t*-tests or one-way ANOVA. We adopted the Šídák multiple comparison test method to control the family-wise error rate (FWER) during multiple comparisons. Logistic regression was used to predict cognitive function status in PE patients and controls. Survival curves were plotted using the Kaplan-Meier method and compared using the log-rank test. Cutoff values were determined through ROC curve analysis.

The Kolmogorov-Smirnov test was used to verify data normality. Normally distributed continuous variables were expressed as mean ± standard deviation, and non-normally distributed variables were expressed as median [25th–75th percentile]. Categorical variables were presented as frequency (percentage). Non-parametric data were compared using the Mann-Whitney U test, while parametric data were analyzed using an unpaired *t*-test (Welch’s correction).

Statistical analyses were performed using SPSS version 27.0 and R Studio software. *P*-value < 0.05 was considered significant. Data analyses were carried out using SPSS 27.0.

## 3 Outcomes

### 3.1 Baseline characteristics

Among the 400 patients with PE and 91 control subjects, age and gender were well-matched. The median age of the PE patients was 69 years, while for the control group it was 70 years, with a *P*-value of 0.331 indicating no significant difference. The number and proportion of males in the patient group were 237 (59.3%), compared to 50 (54.9%) in the control group. The median body mass index (BMI) was 24.17 for the patients and 24.91 for the controls. The educational levels were relatively balanced between the two groups. The comorbidities included hypertension, diabetes, and heart failure and cerebrovascular accidents, with prevalence rates of 38.03%, 9.82%, and 4.13% in the patient group, respectively, and 45.05%, 6.59%, and 1.10% in the control group, respectively. It should be noted that some patients and controls had more than one comorbidity. The main risk factors for venous thromboembolism (VTE) in this study were recent surgery, affecting 187 patients (36.74%) in the PE group, and a history of deep vein thrombosis (DVT), affecting 103 patients (20.24%) in the PE group.

The biochemical indices for the PE patients were as follows: the median D-Dimer level was 145.00 ng/ml, creatinine was 66.00 μmol/L, NT-proBNP was 193.70 pg/mL, and Creatine Kinase-MB (CKMB) was 1.29 ng/mL. Using the Simplified Pulmonary Embolism Severity Index (sPESI) for risk stratification, among the 400 patients, 134 (33.5%) were classified as low risk (sPESI < 1), and 266 (66.5%) were classified as moderate to high risk (sPESI ≥ 1). Additionally, out of all the patients, 65 experienced recurrence. There was no significant difference about demographic data between the two groups (Table 1).

**TABLE 1:** Baseline characteristics in patients with pulmonary embolism and control individuals.

Variables	Pulmonary embolism (*n* = 400)	Control (*n* = 91)	*P*-value
Age, yr	69.00 (65.00–74.00)	70.00 (67.00–73.00)	0.331
Male/female, *n*	237/163	50/41	0.158
BMI, kg/m^2^	24.17 (22.04–26.06)	24.91 (22.83–27.55)	0.100
Education*	3.00 (2.00–4.00)	3.00 (3.00–4.00)	0.132
**Comorbidities,† *n* (%)**			
Hypertension	116 (38.03)	41 (45.05)	0.229
Diabetes	50 (9.82)	6 (6.59)	0.893
Cordis and cerebral accidents	21 (4.13)	1 (1.10)	0.276
**Risk factors for VTE, *n* (%)**			
Recent surgery	187 (36.74)	–	–
History of DVT	103 (20.34)	–	–
**Biochemical indexes**			
D-Dimer, ng/ml	145.00 (3.36–607.00)	–	–
Creatinine, μmol/L	66.00 (55.52–76.15)	–	–
NT-ProBNP, pg/mL	193.70 (67.40–840.80)	–	–
CKMB, ng/mL	1.29 (0.85–2.13)	–	–
Risk level (low risk/high risk)	134/266	–	–
Recurrence/non-recurrent	65/335	–	–

BMI, body mass index; NT-proBNP, N-terminal pro B-type natriuretic peptide; CKMB, Creatine Kinase-MB. Values are means (±SD), medians (interquartile range), or *n* (%).

*The degrees of education, 1 = illiteracy; 2 = primary schools; 3 = junior middle school; 4 = high school or technical secondary school; 5 = junior college; 6 = university; 7 = master’s degree or above.

†Comorbidities only include hypertension, diabetes, and Cordis and cerebal accidents.

### 3.2 Association between cognition and PE

**FIGURE 1:**
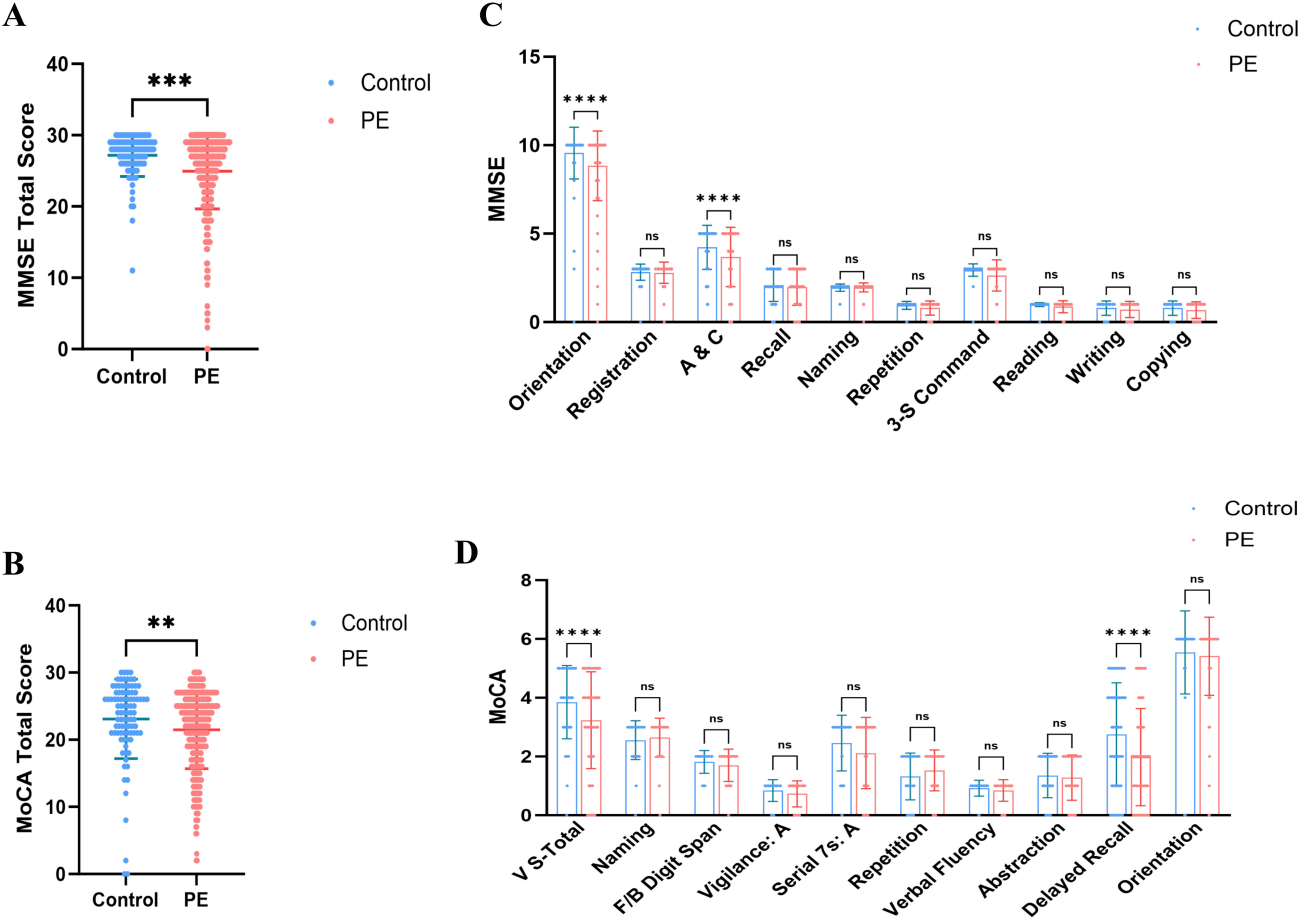
Cognitive dysfunction in patients with PE and control subjects. **(A)** MMSE total scores in patients with PE and control subjects. **(B)** MoCA total scores in patients with PE and control subjects. **(C)** The details of MMSE in patients with PE and control subjects. **(D)** The details of MoCA in patients with ILD and control subjects. Statistical analyses were conducted using Mann-Whitney U test; multiple comparisons and performed tests using the Šídák method. **Highly significant (*P* < 0.01), ***Extremely significant (*P* < 0.001), ****Ultra-significant (*P* < 0.0001).

**FIGURE 2:**
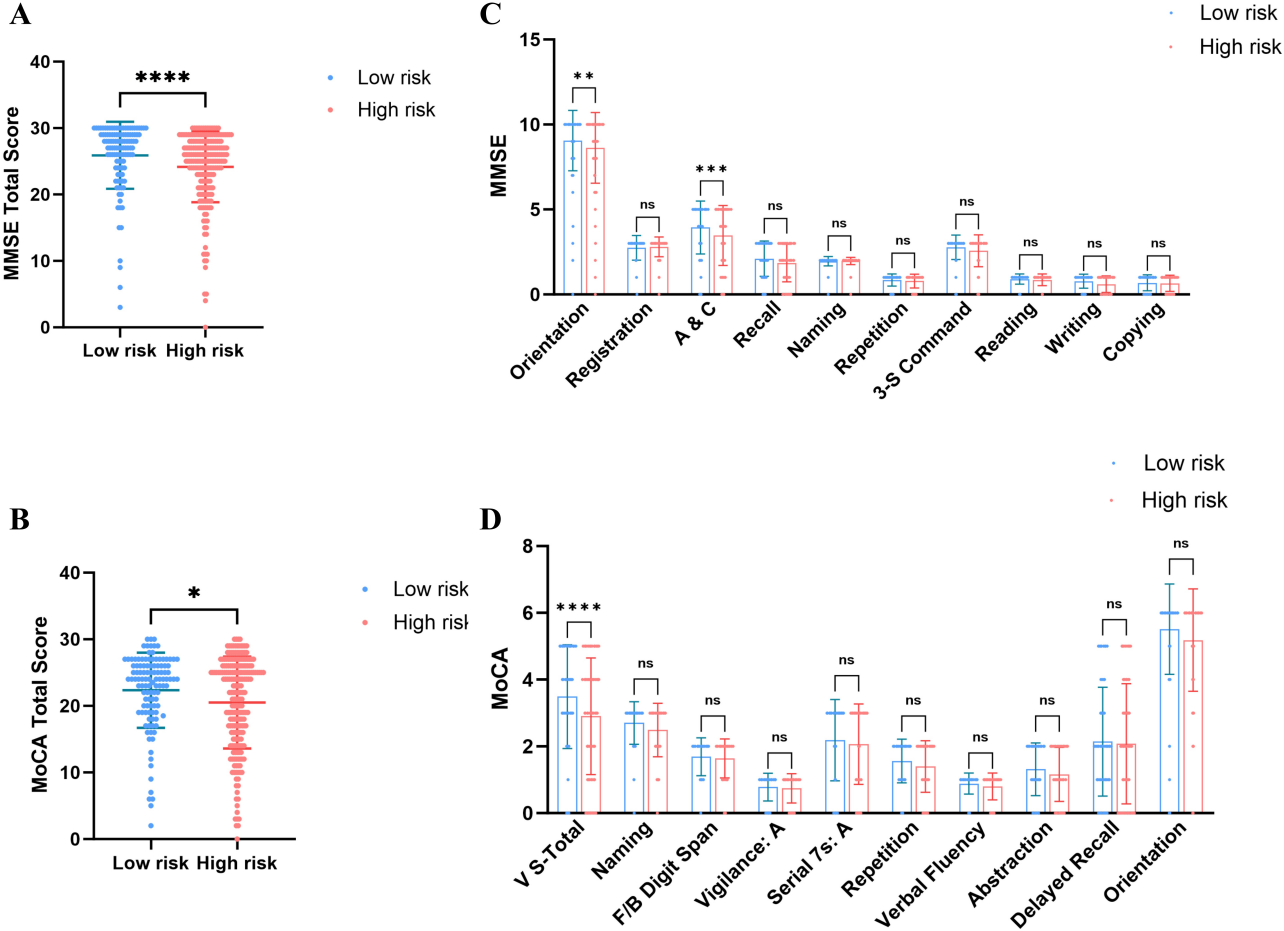
Cognitive dysfunction in patients with PE of low risk and High risk. **(A)** MMSE total scores in patients with PE of low risk and High risk. **(B)** MoCA total scores in patients with PE of low risk and High risk. **(C)** The details of MMSE in patients with PE of low risk and High risk. **(D)** The details of MoCA in patients with PE of low risk and High risk. Statistical analyses were conducted using Mann-Whitney U test; multiple comparisons and performed tests using the Šídák method. *A significant difference (*P* < 0.05), **Highly significant (*P* < 0.01), ***Extremely significant (*P* < 0.001), ****Ultra-significant (*P* < 0.0001).

**FIGURE 3:**
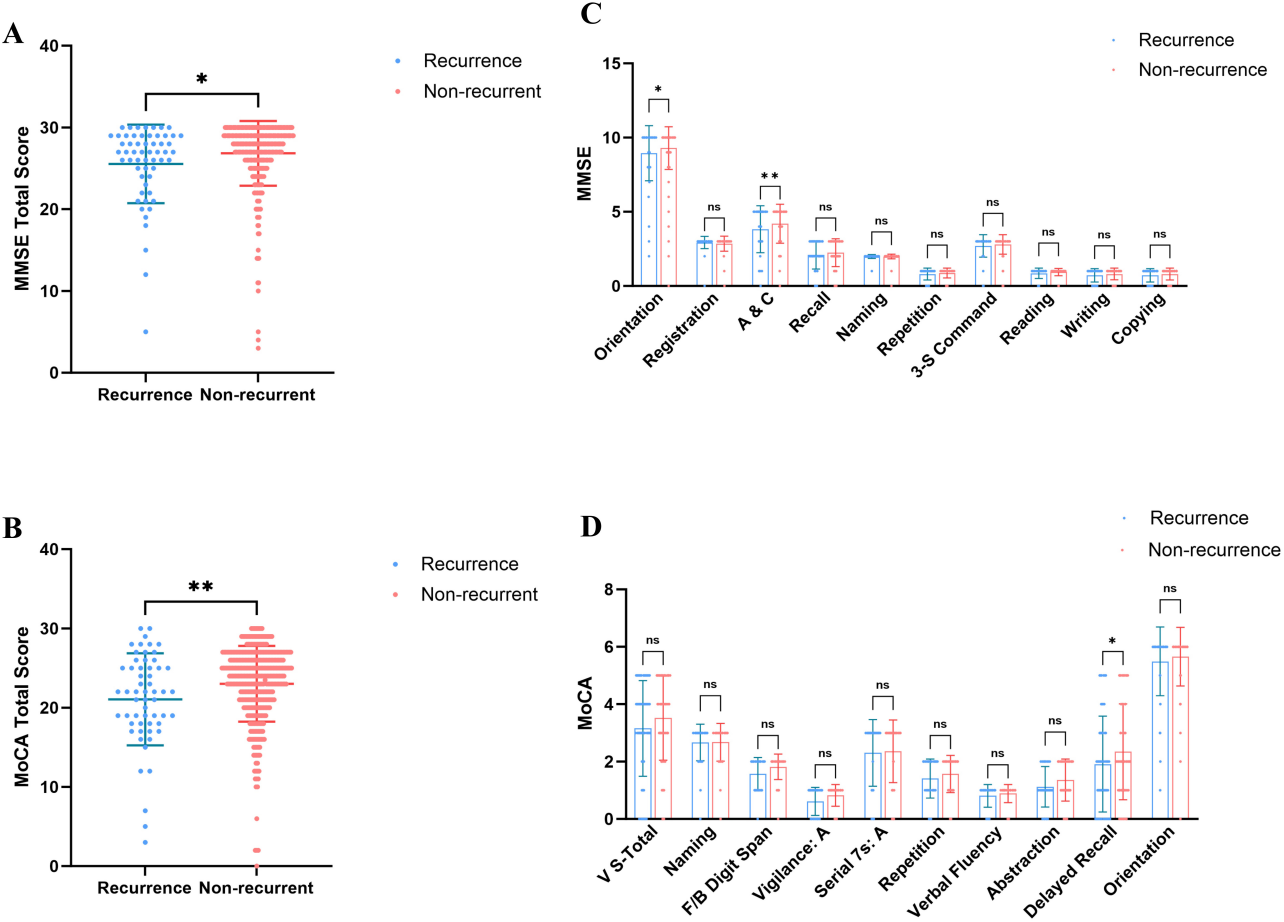
Cognitive dysfunction in patients recurrence and non-recurrent in PE. **(A)** MMSE total scores in patients recurrence and non-recurrent in PE. **(B)** MoCA total scores in patients recurrence and non-recurrent in PE. **(C)** The details of MMSE in patients recurrence and non-recurrent in PE. **(D)** The details of MoCA in patients recurrence and non-recurrent in PE. Statistical analyses were conducted using Mann-Whitney U test; multiple comparisons and performed tests using the Šídák method. *A significant difference (*P* < 0.05), **Highly significant (*P* < 0.01).

Regarding overall cognitive status, PE patients exhibited significantly lower total MMSE and MoCA scores than healthy controls (Figures 1A, B), indicating that PE is associated with widespread cognitive impairment. Sub-item analysis revealed significant declines in Orientation and Attention/Calculation (A&C) (MMSE), as well as Visuoconstructional Skills-Total and Delayed Recall (MoCA) (Figures 1C, D). High-risk PE patients demonstrated significantly lower total MMSE and MoCA scores than low-risk patients (Figures 2A, B), with significant reductions in the sub-items of orientation and A&C (MMSE) and Visuoconstructional Skills-Total (V-S total) (MoCA) (Figures 2C, D). Recurrent PE patients had significantly lower total MMSE and MoCA scores than non-recurrent patients (Figures 3A, B), with significant declines in the sub-items of orientation and A&C (MMSE) and Delayed Recall (Figures 3C, D).

**FIGURE 4:**
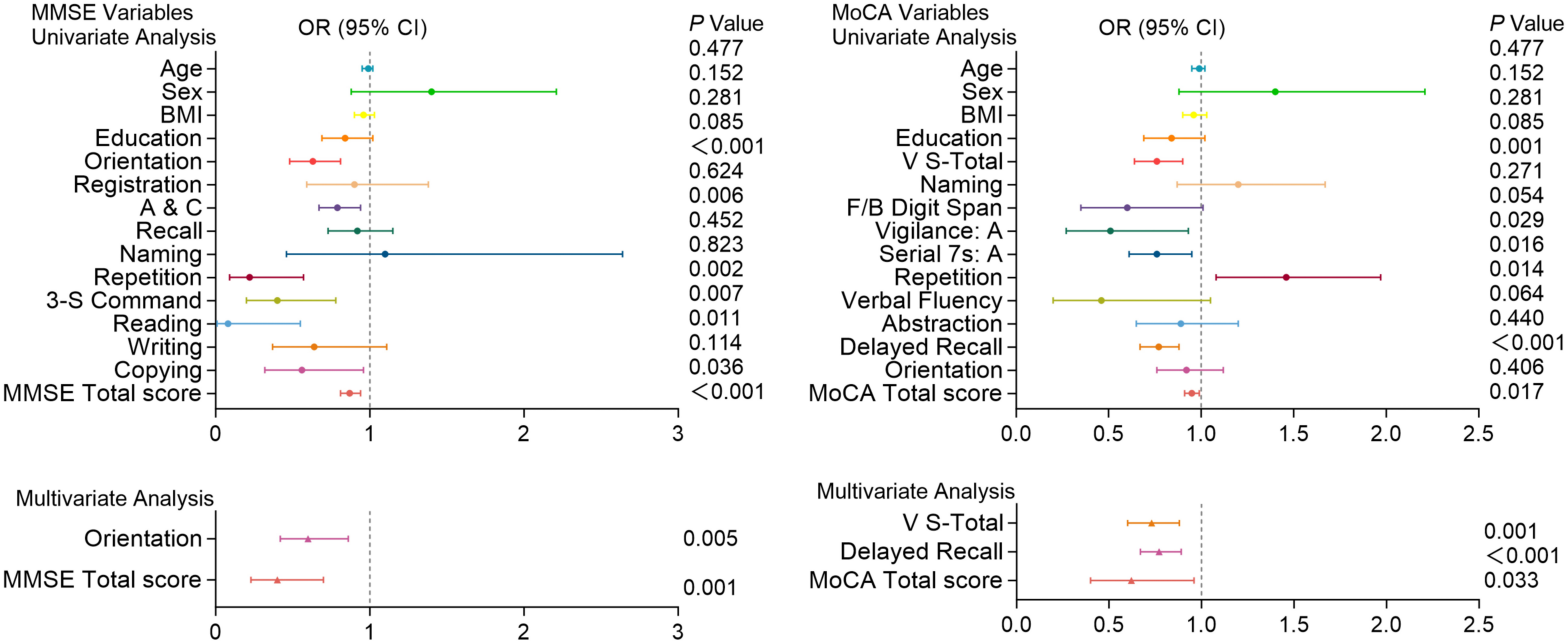
Logistic regression analysis of pulmonary embolism with cognitive function test scores. Univariate and multivariate analyses of MMSE and MoCA variables in patients with and without pulmonary embolism. Factors with *P* < 0.05 in the univariate analysis were included in the multivariate analysis. Including Orientation, A&C, Repetition, 3-S Command, Reading, Copying, and total scores of the MMSE; V-S total, Attention, Serial 7s: A, Delayed recall, and total scores of the MoCA. OR (Odds Ratio) with 95% confidence intervals (CI) and corresponding *P*-values are presented for each variable. Significant associations (*P* < 0.05) are highlighted, indicating that certain cognitive functions are more impaired in patients with pulmonary embolism compared to those without.

**FIGURE 5:**
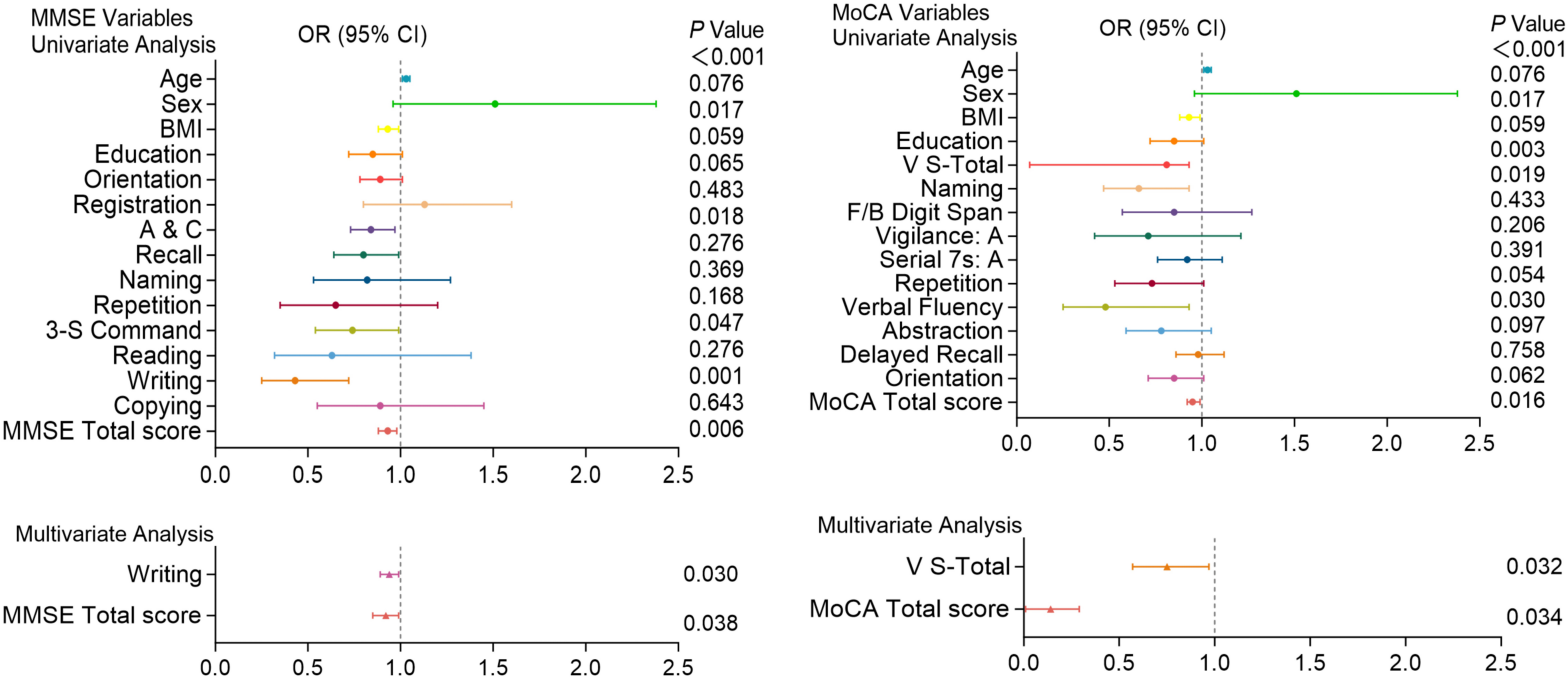
Logistic regression analysis of pulmonary embolism with cognitive function test scores. Univariate and multivariate analyses of MMSE and MoCA variables in patients with and without pulmonary embolism. Factors with *P* < 0.05 in the univariate analysis were included in the multivariate analysis. Including A&C, 3-S Command, writing and total scores of the MMSE; V-S total, naming, verbal fluency and total scores of the MoCA. OR (Odds Ratio) with 95% confidence intervals (CI) and corresponding *P*-values are presented for each variable. Significant associations (*P* < 0.05) are highlighted, indicating that certain cognitive functions are more impaired in patients with pulmonary embolism compared to those without.

**FIGURE 6:**
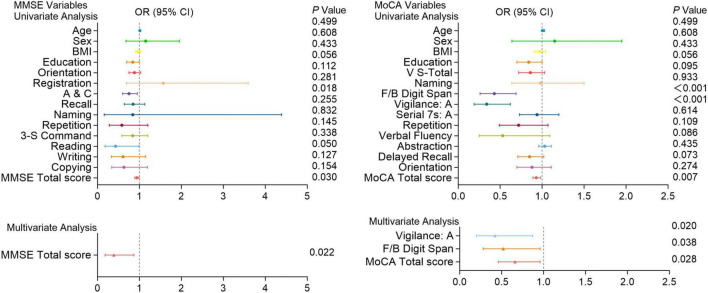
Cognitive function test scores in patients with and without recurrent pulmonary embolism. Univariate and multivariate analyses of MMSE and MoCA variables in patients with and without recurrent pulmonary embolism. Factors with *P* < 0.05 in the univariate analysis were included in the multivariate analysis. Including A&C and total scores of the MMSE; F/B Digit Span, vigilance: A and total scores of the MoCA. OR (Odds Ratio) with 95% confidence intervals (CI) and corresponding *P*-values are shown for each variable. Significant associations (*P* < 0.05) are indicated, highlighting cognitive functions that are more impaired in patients with recurrent pulmonary embolism compared to those without.

Figures 4–6 present the results of the logistic regression analysis. In the univariate analysis, Orientation, A&C, Repetition, three-step command (3-S Command), Reading, Copying, and total scores of the MMSE; V-S total, vigilance: A, Serial subtraction of 7 (Serial 7s: A), Delayed recall, and total scores of the MoCA were significantly associated with the occurrence of PE (*P* < 0.05). Multivariate analysis further confirmed that Orientation (*P* = 0.005) and total score (*P* = 0.001) of the MMSE; V-S total (*P* = 0.001), Delayed recall (*P* < 0.001), and total score (*P* = 0.033) of the MoCA are independent predictors of the occurrence of Figure 4.

In univariate analysis, A&C, 3-S Command, writing and total scores of the MMSE; V-S total, naming, verbal fluency and total scores of the MoCA were significantly associated with the risk of PE (*P* < 0.05). Multivariate analysis further revealed that Writing ability (*P* = 0.030) and total score (*P* = 0.038) of the MMSE, as well as V-S total (*P* = 0.032) and total score (*P* = 0.034) of the MoCA are independent predictors of high-risk PE (Figure 5).

Moreover, In univariate analysis, A&C and total scores of the MMSE; Digit memory Span (F/B Digit Span), vigilance: A and total scores of the MoCA were significantly associated with the recurrence of PE (*P* < 0.05). Multivariate analysis shows the total score of the MMSE (*P* = 0.022); vigilance: A (*P* = 0.020), F/B Digit Span (*P* = 0.038), and total score (*P* = 0.028) of the MoCA are independent predictors of PE recurrence (Figure 6).

### 3.3 The associations of cognition with risk and recurrence rate of PE

**FIGURE 7:**
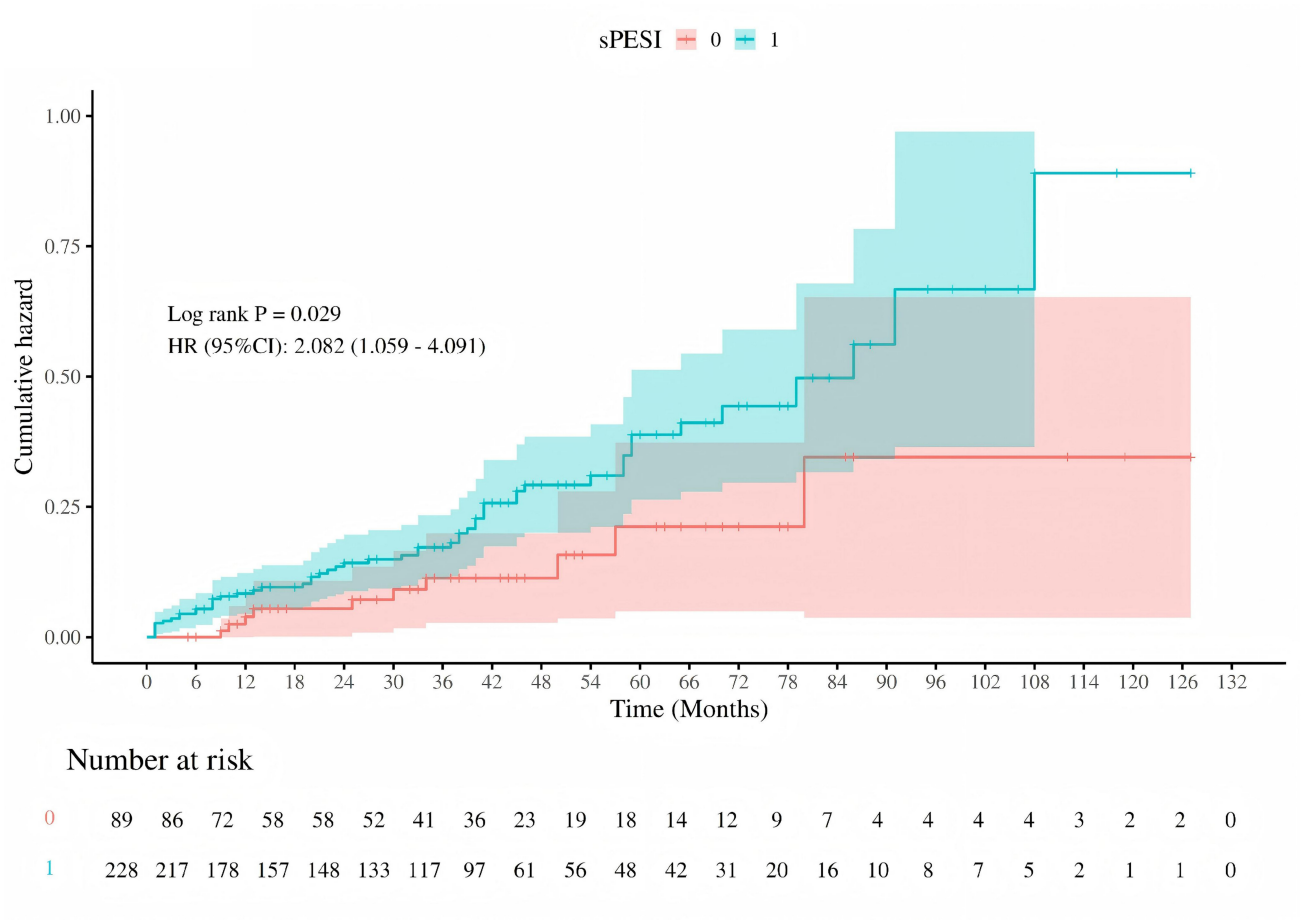
Cumulative hazard of pulmonary embolism recurrence by sPESI risk stratification. Kaplan-Meier survival curve comparing the cumulative hazard of pulmonary embolism recurrence between low-risk (sPESI = 0, red) and high-risk (sPESI = 1, blue) groups. The log-rank test *P*-value is 0.029, and the hazard ratio (HR) with 95% confidence interval (CI) is 2.082 (1.059–4.091), indicating a significantly higher risk of recurrence in the high-risk group. The number of patients at risk is shown at the bottom of the graph.

**FIGURE 8:**
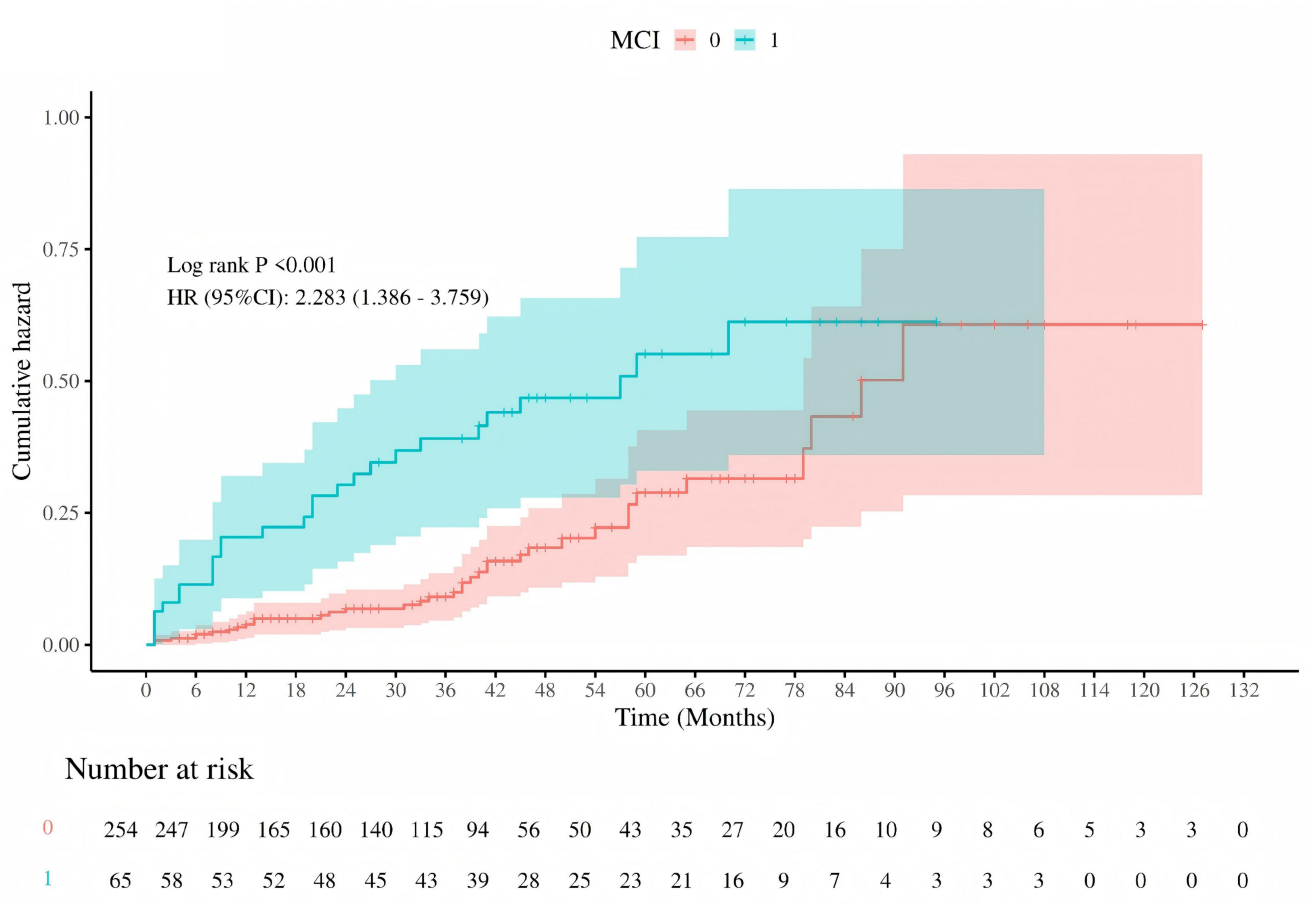
Cumulative hazard of recurrent pulmonary embolism by MCI status. Kaplan-Meier survival curve depicting the cumulative hazard of recurrent pulmonary embolism in patients with (MCI = 1, blue) and without (MCI = 0, red) mild cognitive impairment (MCI). The log-rank test *P*-value is less than 0.001, and the hazard ratio (HR) with 95% confidence interval (CI) is 2.283 (1.386–3.759), indicating a significantly higher risk of recurrence in patients with MCI. The number of patients at risk is shown at the bottom of the graph.

Kaplan-Meier survival curves were utilized to investigate the association between MMSE and MoCA with clinical deterioration events in patients with PE. The Kaplan-Meier survival curves indicated that patients at high risk for PE had a higher probability of recurrence (log-rank *P* = 0.029, Figure 7), and among those who recurred, there was a higher probability of developing mild cognitive impairment (MCI) (log-rank *P* < 0.001, Figure 8).

**FIGURE 9:**
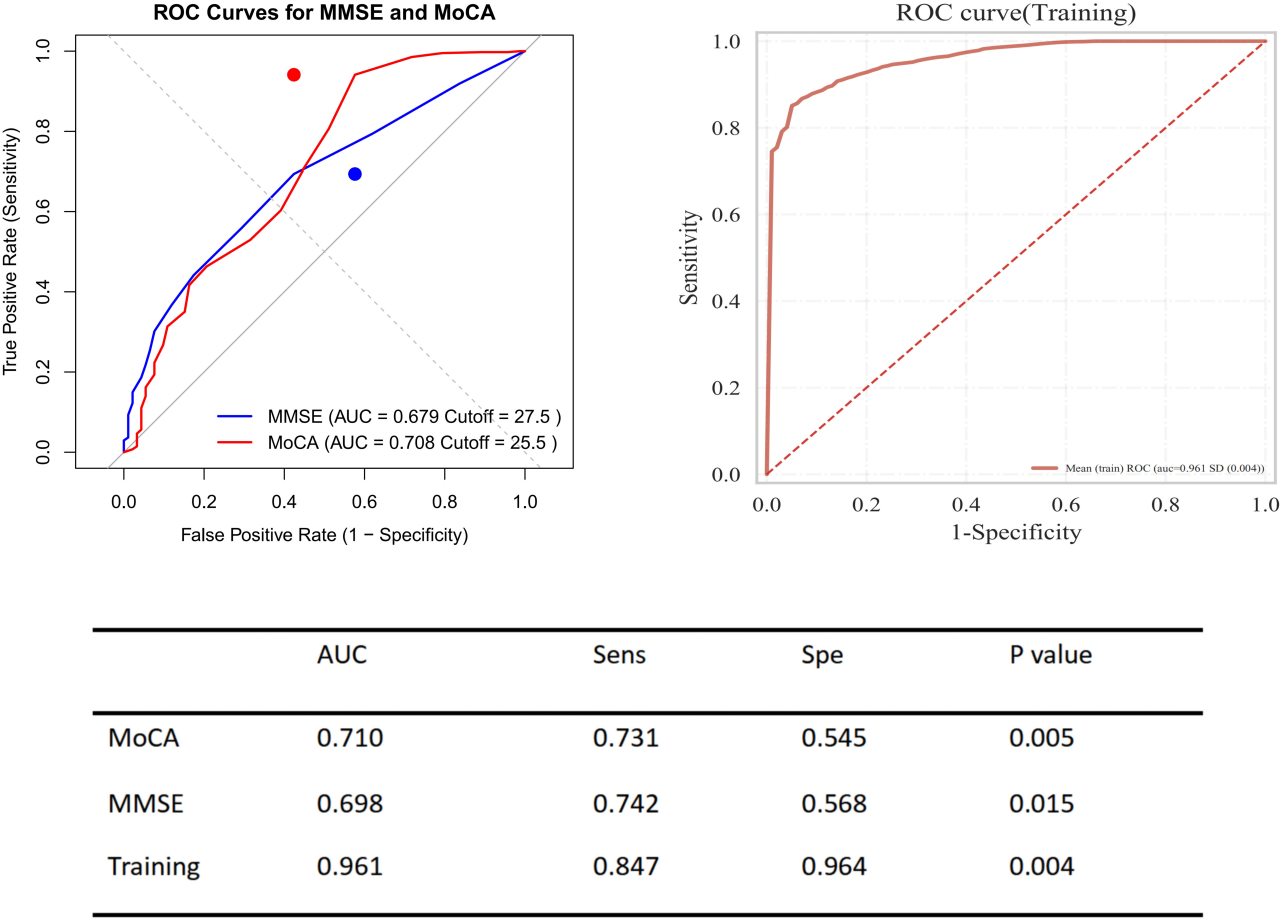
Receiver operating characteristic (ROC) curves for predicting presence of pulmonary embolism using MMSE and MoCA. Left panel displays ROC curves for MMSE (blue) and MoCA (red) to predict the presence of pulmonary embolism, with AUC values of 0.679 (cut-off = 27.5) and 0.708 (cut-off = 25.5), respectively. Right panel shows the ROC curve for the training model with an AUC of 0.961. The table summarizes performance metrics including AUC, sensitivity (Sens), specificity (Spe), and *P*-values for both cognitive tests and the training model.

In patients with PE, the area under the ROC curve for the MMSE was 0.679 (sensitivity 74.2%, specificity 56.8%, *P* = 0.015); for the MoCA, it was 0.710 (sensitivity 73.1%, specificity 54.5%, *P* = 0.005), compared with the healthy control group. Additionally, the combined predictive model of the two scores significantly improved the prediction of disease risk associated with PE, with an area under the ROC curve of 0.961 (sensitivity 84.7%, specificity 96.4%, *P* = 0.004) (Figure 9). Based on the ROC analysis, patients with lower MMSE and MoCA total scores (MMSE < 27.5, MoCA < 25.5) had a higher rate for events.

## 4 Discussion

Existing studies have reported that PE can lead to acute ischemic encephalopathy, presenting with cognitive abnormalities such as confusion, disorientation, and reduced attention. Additionally, it has been noted that 2 years after a pulmonary embolism, some patients experience persistent functional impairments, including dyspnea, reduced exercise tolerance, and decreased quality of life. Although cognitive function was not directly assessed in these patients, the psychological dimension scores of quality-of-life questionnaires (such as EQ-5D-5L) revealed significant self-reported difficulties in attention and memory, indicative of cognitive impairment (21).

To date, reports have primarily focused on the identification of related symptoms, lacking large-scale patient cohorts and cognitive function evaluations. In this study, we included 400 patients with PE, matched with controls by age and gender, and assessed their cognitive function using the MMSE and MoCA cognitive function scales. In addition to routine diagnostic parameters, comorbidities, and laboratory tests, we evaluated the patients’ risk stratification and monitored whether they experienced recurrence, comprehensively exploring the relationship between PE occurrence, risk stratification, recurrence, and cognitive function.

In the baseline table of this study, it can be observed that both the PE patients and the control group cohort have comorbidities such as diabetes, hypertension, and cardiovascular and cerebrovascular diseases, and the overall age is relatively high. The interaction of PE, comorbidities, and advanced age makes patients relatively more frail, which to some extent increases the severity of cognitive impairment. Some studies have explored the association between frailty and cognitive function through a pooled analysis of data from six prospective cohort studies and found a significant correlation between frailty and cognitive decline (9). Other studies have also pointed out that inflammation plays an important mediating role between frailty and cognitive impairment in older adults (24). Although the results were not displayed, we found in our study that some patients in the control group had low MMSE and MoCA scores, which is consistent with the findings of other existing studies. This may be due to cognitive decline caused by age and related comorbidities. Therefore, in our study, we included patients with comorbidities in both the control and PE groups to balance the baseline of the patient cohorts.

In this study, we found that patients with PE occurrence, moderate-to-high risk, and recurrence had significantly lower total scores on the MMSE and MoCA, as well as certain sub-item scores. Logistic regression analysis indicated that these total and sub-item scores could independently predict the occurrence of events. Regarding total scores, the MMSE and MoCA total scores were independently associated with the occurrence and prognosis of PE. This suggests that the observed cognitive deficits are not random events but may reflect underlying pathologies related to PE, such as hypoxia, inflammation, and microembolism. This underscores the importance of incorporating cognitive assessment as part of the comprehensive evaluation of PE patients. Early identification of cognitive impairment through tools like the MMSE and MoCA can facilitate early intervention and management strategies aimed at improving cognitive outcomes and potentially reducing the risk of PE recurrence. This highlights the clinical value of integrating cognitive screening into routine PE care.

In the sub-items of the two scales, we found that orientation, visuospatial transformation ability, and delayed recall in PE patients were independently associated with the occurrence of PE. This could be due to PE causing pulmonary artery obstruction, leading to acute or chronic hypoxia. Hypoxia can widely affect the metabolic activity of the cerebral cortex, especially in regions that are sensitive to hypoxia, such as the hippocampus and prefrontal cortex (19). These regions are closely related to orientation, visuospatial ability, and memory function. For example, the hippocampus is extremely sensitive to hypoxia, and brief ischemia and hypoxia can lead to functional impairment, thereby affecting delayed recall ability.

In addition to genetic risk factors, previous history of venous thrombosis, surgical trauma, malignancy, cardiovascular and chronic diseases; inadequate duration of anticoagulation therapy; age and NT-proBNP levels can all influence the risk stratification and prognosis of PE patients (5, 25). In this study, we found that writing ability, visuospatial transformation ability, digit span memory, and alertness are independently associated with the recurrence and high risk of PE. In our patient cohort, these aforementioned risk factors for recurrence are commonly present. Therefore, under the complex interplay of these factors, compared to the previous orientation, these sub-items involve complex cognitive activities that require the coordination of language comprehension, motor planning, visual feedback, and fine motor control. Their neural basis involves multiple brain regions such as the frontal lobe (e.g., Broca’s area), parietal lobe, and basal ganglia (26). The impairment of these abilities may reflect more widespread brain network dysfunction caused by PE, especially in the integration of language and motor functions. Higher-order cognitive dysfunction is more prominent in patients with high-risk and more severe PE. The overall cognitive decline is more significant in high-risk PE patients.

In addition to performing logistic regression predictions for the occurrence, risk stratification, and recurrence of PE based on the total scores and sub-item factors of the two scales, further Kaplan-Meier (KM) analysis and receiver operating characteristic (ROC) curve analysis were conducted. However, given that a greater number of continuous variables can provide richer information, higher statistical sensitivity and specificity, clearer survival analysis results, stronger clinical significance and interpretability, as well as more continuous and comparable data, only the total scores of the two scales were selected for KM and ROC curve analysis in this study. The analysis indicated that high-risk PE patients have a higher recurrence rate, and patients with mild cognitive impairment are at greater risk of recurrence. The combined predictive value of the MMSE and MoCA scales (MMSE < 27.5 and MoCA < 25.5) was highlighted.

Compared to previous studies that were mostly based on clinical case reports, this study evaluated the cognitive function of PE patients using the MMSE and MoCA, further exploring the relationship between their risk stratification, recurrence status, and cognitive impairment. We found that patients with poorer cognitive function have a higher likelihood of developing pulmonary embolism (PE) and worse prognosis. This may be closely related to the molecular mechanisms of the disease previously reported, such as hypoxia, microembolism, and inflammatory responses. This suggests that cognitive dysfunction may exist in patients with PE, providing a basis for clinical cognitive screening in patients with PE. It further indicates that patients with PE are a potential population for cognitive rehabilitation and should be identified and intervened as early as possible.

## 5 Limitations

We can observe that the control group cohort in this study is significantly smaller than the patient group. However, matching by age and gender allows for further statistical analysis. As this is a retrospective study, the next step should involve expanding the control group to conduct a larger prospective cohort study. Secondly, due to the lack of matching between the patients’ admission time, diagnosis time, and cognitive function assessment time, some patients had already been diagnosed with pulmonary embolism (PE) before admission to our hospital. We decided to conduct assessments using two scales for all patients after admission. The potential mismatch between the diagnosis time of pulmonary embolism and the cognitive testing may lead to confusion in interpreting cognitive impairment as being caused by pulmonary embolism. This represents an objective limitation. Further optimization of screening criteria is needed to select patients with more closely aligned diagnosis and assessment times for detailed analysis. Thirdly, the MMSE and MoCA scales are relatively subjective, and this study lacks objective indicators of cognitive impairment such as brain MRI or PET-CT (27). Future studies should incorporate more objective measures to enhance the sensitivity and specificity of the conclusions. Lastly, this study has only revealed a phenomenon at the clinical data analysis level and has not conducted validation or exploration at the animal or cellular level. Subsequent research should delve deeper into these aspects.

## Data Availability

The raw data supporting the conclusions of this article will be made available by the authors, without undue reservation.
